# Bibliometric and visual analysis of Kawasaki disease in children from 2012 to 2022

**DOI:** 10.3389/fped.2023.1142065

**Published:** 2023-07-27

**Authors:** Zhengjiu Cui, Fei Luo, Jinjuan Wang, Juanjuan Diao, Yueli Pan

**Affiliations:** ^1^ First College of Clinical Medicine, Shandong University of Traditional Chinese Medicine, Jinan, China; ^2^ Department of Pediatrics, Affiliated Hospital of Shandong University of Traditional Chinese Medicine, Jinan, China

**Keywords:** kawasaki disease, children, bibliometric analysis, visual analytics, VOSviewer, CiteSpace

## Abstract

**Background:**

In recent years, the incidence of Kawasaki disease among the pediatric population has experienced a significant increase. With complications mainly affecting the cardiovascular system, Kawasaki disease has received widespread attention from scholars worldwide. Numerous articles on Kawasaki disease in children have been published far. However, there is a lack of studies that use visualization methods to perform a bibliometric analysis of the relevant literature. This study aims to obtain overall information on the output characteristics of publications on childhood Kawasaki disease between 2012 and 2022 through bibliometric analysis, identify research hotspots and frontiers, and provide new ideas and references for future clinical and scientific research.

**Methods:**

Literature meeting the inclusion criteria was screened from the Web of Science Core Collection, PubMed, and Scopus databases. Visual analysis of the literature by country, institution, journal, author, keywords, and references was performed using Citespace (6.1.R6), VOSviewer (1.6.18), and the online bibliometric website (https://bibliometric.com/).

**Results:**

A total of 4,867 eligible publications were included. The number of annual publications is generally rising, rapidly increasing since 2019. Among countries and institutions, China and KAOHSIUNG CHANG GUNG MEMORIAL HOSPITAL have the highest output of articles. With 104 publications, Ho-Chang Kuo has a high impact in the field of KD. The most cited author is Jane W. Newburger. The most prolific journal is FRONTIERS IN PEDIATRICS. CIRCULATION is the most frequently co-cited journal. The most popular keyword in frequency and centrality is “immunoglobulin”. The reference with the highest burst intensity was Verdoni L, LANCET, 2020.

**Conclusion:**

Kawasaki disease in children remains a hot topic among pediatricians worldwide and is receiving increasing attention. We innovated the “national-institutional-journal” model, which promotes further international cooperation in this field. The hot topics in the field of pediatric KD are “KD pathogenesis”, “immunoglobulin resistance and complementary therapy”, and “cardiovascular complications”. Frontiers include disease-related (“multisystem inflammatory syndrome”, “coronavirus disease 2019”, “hypotension”), treatment-related (“procalcitonin”, “ anakinra”), and pathogenesis (“polymerase chain reaction”).

## 1. Introduction

Kawasaki disease (KD), also known as mucocutaneous lymph node syndrome, is an acute systemic vasculitis of unknown etiology that often affects children under five years of age but is not limited to children (1, 2). The incidence is about 1.5 times higher in males than females (3). It was first reported by a Japanese pediatrician, Tomisaku Kawasaki, in 1967 (2). Currently, KD is prevalent in more than 60 countries worldwide. However, the incidence varies by geographic location and season, with East Asian countries such as Japan, Korea, and China having a prevalence 10–30 times higher than that of the United States and Europe, and peaks between late winter and spring and summer (4, 5). The American Heart Association further classifies KD into complete and incomplete KD, which states that complete KD has all the typical clinical features (fever, rash, oral mucosal changes, bilateral non-exudative conjunctivitis, enlarged cervical lymph nodes, and terminal edema of the extremities) (6). Although KD is self-limiting, it remains the most common cause of acquired heart disease in children in the United States. Approximately 30% of patients with untreated acute phase KD develop coronary artery dilation (CAD) and even severe coronary artery aneurysms (CAA) (7). The cause of death in KD is primarily cardiac sequelae concentrated in the 15–45 days after fever (8). Incomplete KD can also involve the digestive (9), neurological (10), genitourinary (11), skeletal-muscular (12), and respiratory (13) systems. The current main standardized treatment for the acute phase of KD is high-dose intravenous immunoglobulin (IVIG) and aspirin, which aim to reduce the inflammatory response, mitigate arterial damage, and prevent thrombosis (14). The rising prevalence and risk of complications have led to more KD-related studies. Since the outbreak of the novel coronavirus disease in 2019, the multisystemic inflammatory syndrome has emerged in pediatric patients with the infection, and its clinical features highly coincide with KD, rendering the discrimination between the two challenging, and scholars pressed to study KD more intensively (15). However, there needs to be standardized correlational and chronological analysis of the global literature on KD in children.

The bibliometric approach is used to explore the underlying knowledge structure contained in the scholarly literature of a field and integrate the visualization results for further analysis, allowing researchers to comprehensively extract quantitative information about the distribution by country, institution, author, and journal, reflecting the characteristics, impact and future trends of published research results in a field (16). Both Citespace and VOSviewer are software tools for constructing and visualizing bibliometric networks. Citespace (17) uses co-citation, co-occurrence, burst detection, and cluster analysis to visualize topics. VOSviewer (18) can construct and visualize co-occurrence networks of important terms extracted from a large body of scientific literature. In recent years, bibliometric and visual analysis has been widely used in the medical field (19–22). Web of Science (WOS) is the world's largest comprehensive multidisciplinary core journal database (23). PubMed is a global biomedical literature retrieval system developed by the U.S. National Library of Medicine (24). Scopus is the world's largest abstract and citation database of peer-reviewed literature (25). To our knowledge, there are no reports of bibliometric analysis pediatric KD through the above three large authoritative databases.

In this study, we used Citespace (6.1.R6), VOSviewer (1.6.18), and the online bibliometric website (https://bibliometric.com/) to statistically analyze and visualize the 2012 to 2022 WOS Core Collection, the PubMed and Scopus databases for literature related to KD in children. Our ultimate goals are mainly to: (i) identify the influential countries, institutions, authors, and journals with outstanding contributions in the field of pediatric KD; (ii) elucidate the current status, hotspots, and future trends of research; and (iii) promote collaborative research among scholars worldwide in the hotspots and bottlenecks of pediatric KD.

## 2. Materials and methods

### 2.1. Data sources

**Figure 1:**
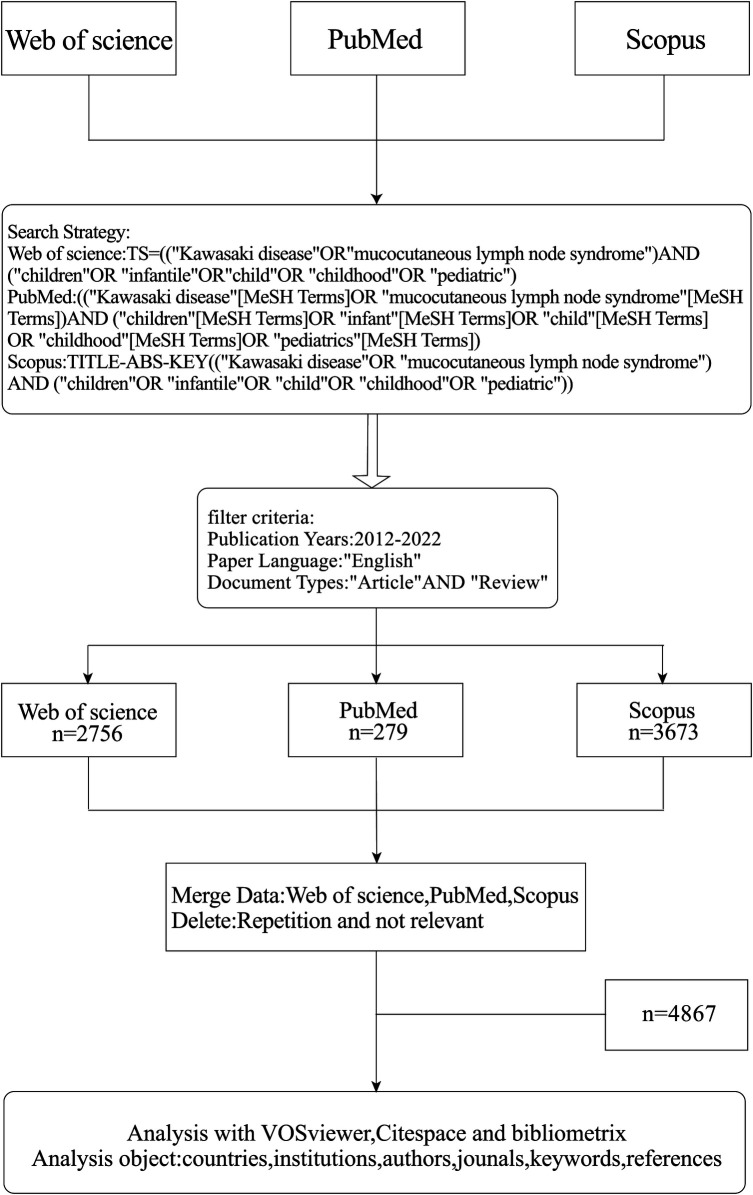
Flowchart of literature collection and selection.

Related literature was retrieved from WOS, PubMed, and Scopus databases on May 29, 2023. The following search terms were used in the subject and keywords: (“Kawasaki disease” OR “mucocutaneous lymph node syndrome”) AND (“children” OR “infantile” OR “child” OR “childhood” OR “pediatric”). The search time range was limited to January 1, 2012, to December 31, 2022. Article types were selected as articles and reviewed papers, excluding non-journal articles. The selected language was “English”. 6,710 articles were collected from the three databases that met the requirements. Two authors (Luo and Wang) independently collected all the data, and the agreement rate was 0.96, which means the data source is accurate and comprehensive. The literature information of the three databases was exported in Txt format, and all data were integrated and adjusted to WOS format uniformly. Zhengjiu Cui and Juanjuan Diao removed duplicate literature and literature not related to the topic. Finally, 4,867 pieces of literature were included in the study, and the specific search flow chart is shown in Figure 1.

### 2.2. Data analysis

We used Excel to compile statistics on the number of annual publications, countries of publication, institutions of publication, and authors of publications. Countries were aggregated by combining Taiwan, Hong Kong, Macao, and People R China into CHINA, Scotland, England, Wales, and Northern Ireland into the United Kingdom. We used the online bibliometric website (https://bibliometric.com/) (26) to visualize the international collaboration between countries. We also used Citespace (6.1.R6) and VOSviewer (1.6.18) to extract essential noun phrases from the titles, abstracts, and keywords of the literature for literature co-word, co-occurrence, and emergent word analysis. We used both software for mapping to obtain the significant contributors and research hotspots in children's KD. In Citespace, the time span was selected as 2012–2022; the time node was set to 1 year; the node type was selected as Country, Keyword, etc.; the node strength defaulted as Cosine, the threshold value was selected as TOP50, the network cropping ribbon was selected as pathfinder algorithm for mapping analysis, and in the analysis of keyword change over time, the VOSviewer was used to analyze countries, institutions, authors, journals, keywords, etc. The data type was selected from WOS, the counting method was set to Full counting, and the word list was added to clean the data, and then the minimum frequency of occurrence of words was set to finally form the graph.

## 3. Results

### 3.1. Trends in the number of publications

**Figure 2:**
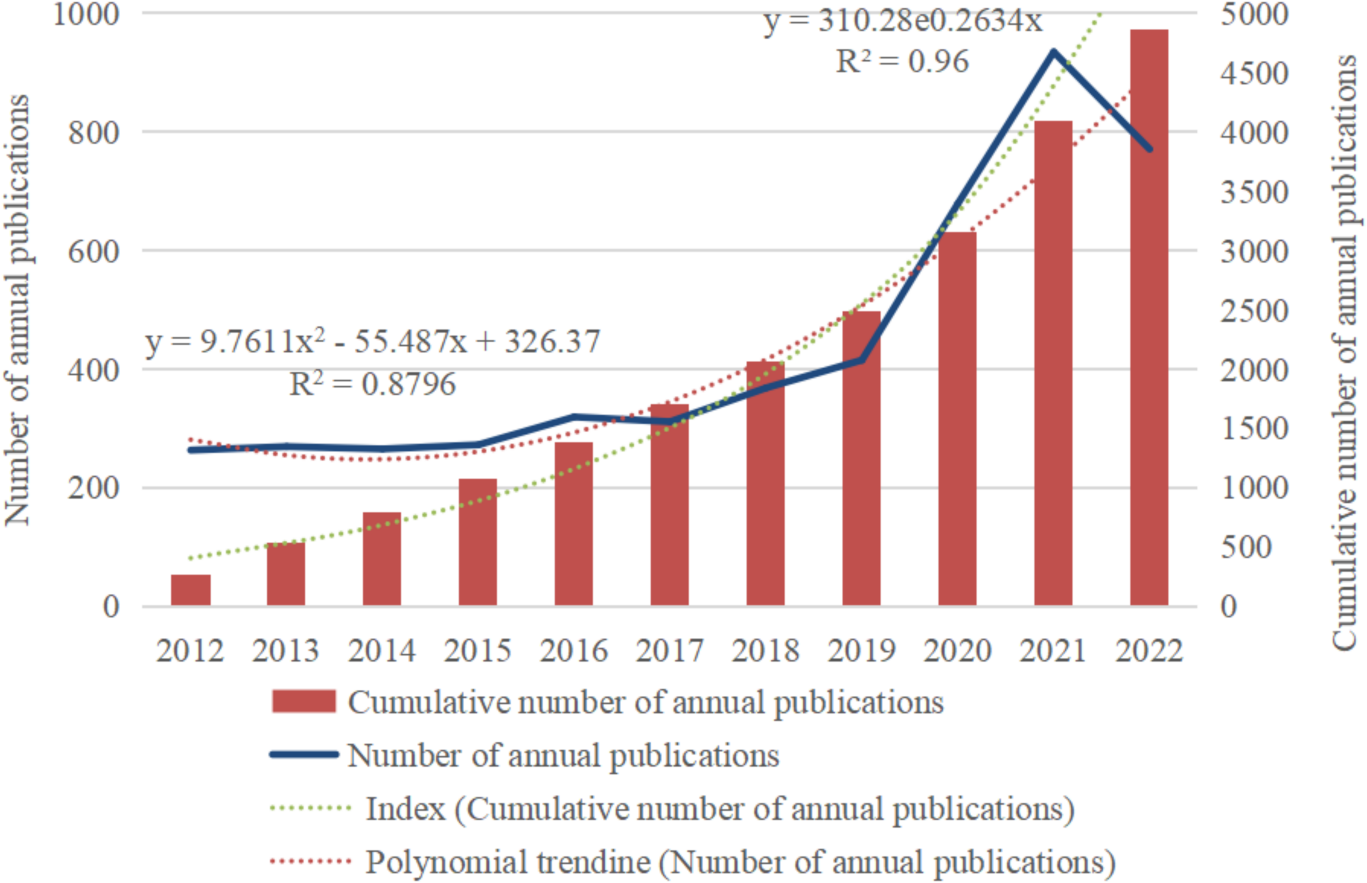
Trend graph of the number of publications on children's KD research (2012–2022).

As shown in Figure 2, the overall upward trend in the number of publications related to children's KD between 2012 and 2022 can be divided into two phases based on the growth rate: the slow fluctuating rise in the number of publications from 2012 to 2019, a period of steady development; and the rapid growth in the number of articles from 2019 to the present, a period of booming prosperity. The polynomial fitting curve in red shows that the correlation coefficient R^2^ between the number of annual publications and the year of publication is 0.8796, and the correlation coefficient R^2^ of the Index curve in green is 0.96, indicating a strong correlation between the number of annual publications, the total number of publications and the year. The number of publications in 2014 and 2017 decreased slightly compared with the previous year, by 4 and 8 articles, respectively. 2020 compared with the previous year. The increase of 264 articles, with a 63.6% YoY growth rate, was the highest in the last decade. 2021 saw a peak in the number of publications in children's KD literature (*n* = 935). As of the search date, the number of publications in 2022 was 771. Considering the lag in publication, it is assumed that the total number of publications in 2022 will maintain a high growth trend. The above data indicate that the field of KD in children has received increasing attention from scientists worldwide in recent years, especially after 2019, when it has become a hot topic with fruitful related research results.

### 3.2. Distribution of countries and institutions

**Figure 3:**
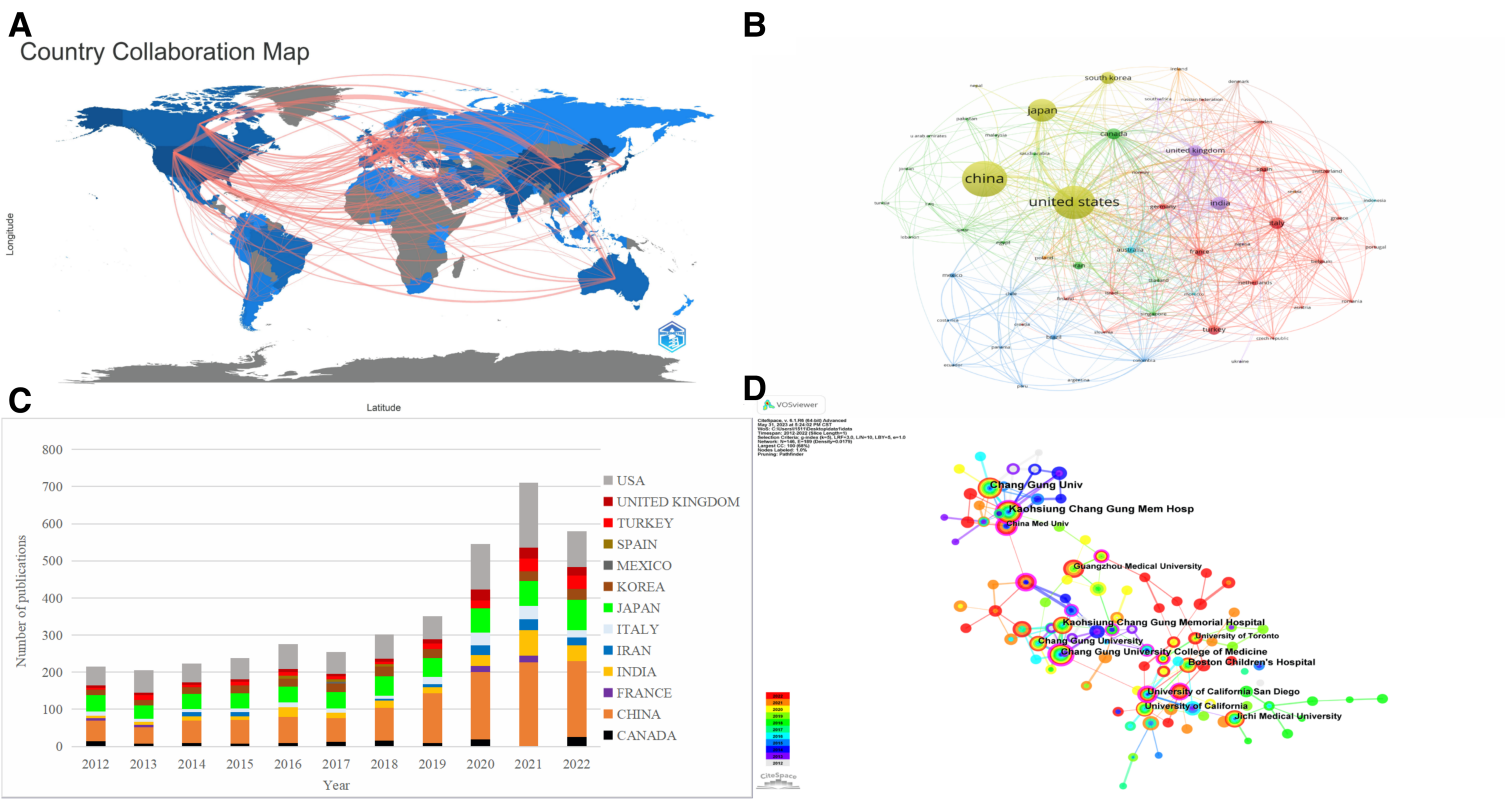
Visualization map of the countries and institutions studying children with KD. (**A**) World Collaborative Relationships Map. (**B**) VOSviewer network map for Countries. (**C**) Trend graph of the top 13 countries to which the authors belong. (**D**) CiteSpace network map for institutions.

According to statistics, the global distribution of research countries and institutions involved in KD in the last decade is broad, but there are uneven characteristics. Figure 3A shows solid collaborative relationships among North America, Europe, and East Asia. The different colors in Figure 3B represent the clusters with a strong association, where the size of the circle indicates the number of publications, and the line's thickness indicates the connection's strength. It can be visualized that among the top five countries in terms of the number of publications, CHINA (*n* = 1,228), UNITED STATES (*n* = 1,103), JAPAN (*n* = 603), and SOUTH KOREA (*n* = 239) are all in the yellow cluster and occupy a central position. Table 1 lists the top 5 countries with the highest centrality, and the centrality index measures the prominence of the network nodes, with UNITED STATES (0.42) being the core of the network, followed by ITALY (0.15), CANADA (0.11), CHINA (0.06), and AUSTRALIA (0.06). Figure 3C plots the trends of the top 13 countries to which the authors belong, and it can be seen that the trends of these countries are roughly the same as the total trend of publications, with CHINA showing significant growth in the number of publications with great potential. The top 5 institutions in terms of volume and centrality are listed in Table 2 and visualized in Figure 3D. KAOHSIUNG CHANG GUNG MEMORIAL HOSPITAL (*n* = 161) published the most papers, followed by Chang Gung University (*n* = 150) and Boston Children's Hospital (*n* = 59). China Medical University had the highest centrality (0.22), followed by Chang Gung University College of Medicine (0.2) and University of California San Diego (0.2), which have purple circles visible in Figure 3D, indicating that it has some influence in the field.

**Table 1:** The top 5 countries with the highest number of publications and highest centrality.

Rank	Countries	Publications	Countries	Centrality
1	CHINA	1,228	UNITED STATES	0.42
2	UNITED STATES	1,103	ITALY	0.15
3	JAPAN	603	CANADA	0.11
4	INDIA	269	CHINA	0.06
5	SOUTH KOREA	239	AUSTRALIA	0.06

**Table 2:** The top 5 institutions with the highest number of publications and highest centrality.

Rank	Institutions	Country	Publications	Institutions	Country	Centrality
1	KAOHSIUNG CHANG GUNG MEMORIAL HOSPITAL	CHINA	161	China Medical University	CHINA	0.22
2	Chang Gung University	CHINA	150	Chang Gung University College of Medicine	CHINA	0.2
3	Boston Children's Hospital	UNITED STATES	59	University of California San Diego	UNITED STATES	0.2
4	Chang Gung University College of Medicine	CHINA	55	China Medical University Hospital	CHINA	0.16
5	Jichi Medical University	JAPAN	53	Boston Children's Hospital	UNITED STATES	0.15

### 3.3. Distribution of authors and co-cited authors

**Figure 4:**
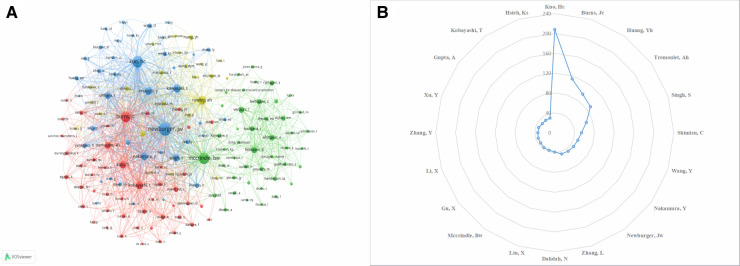
Analysis of the authors. (**A**) Visualization of co-cited authors. (**B**) Author publications radar chart.

The analysis of authors and co-cited authors will help to strengthen the collaboration and identify the main contributors in the field of KD in children. Table 3 lists the top 5 most published and cited authors, mainly from the US and China. Ho-Chang Kuo was the author with the highest number of published articles, with 208, followed by Jane C. Burns (114 articles), Ying-Hsien Huang (96 articles), Adriana H. Tremoulet (89 articles) and Surjit Singh (67 articles). We created a radar plot of the authors' publication volume (Figure 4B) to show a more visual comparison of the number of publications by the top 20 authors, from which it is clear that Ho-Chang Kuo is leading the field and is the leading researcher. There are ten authors from China and four authors from the US, confirming the correlation between authorship and country in terms of the number of publications, reflecting that the number of scholars determines the research output of KD for children in essential countries and that countries with more publications have more scientists involved than the output of academic clusters of very few individuals. There are ten authors with more than 40 publications who have achieved some academic results in the field and contributed to the progress of pediatric KD research. Co-citation analysis refers to when two authors or papers are cited by a third author or paper simultaneously, the two authors or papers have a co-citation relationship, the node size indicates the number of co-citations, and the thickness of the connecting line indicates the degree of citation. The degree of citation is a crucial indicator of an author's contribution. Table 3 shows that Jane W. Newburger (*n* = 770), McCrindle, BW (*n* = 566), Ho-Chang Kuo (*n* = 564), Jane C. Burns (*n* = 564), and Anne H. Rowley (*n* = 564) have the highest number of co-citations. Figure 4A validates this and shows that they represent the core authors of the different clusters leading the development of the field and that the clusters also show a strong connection with each other. These results indicate a strong interest in KD-related research among scholars worldwide and that a sizeable research landscape has been formed led by prominent leading researchers.

**Table 3:** Top 5 ranked authors and co-cited authors.

Rank	Authors	Publications	Country	Co-Cited Authors	Co-Citations	Country
1	Ho-Chang Kuo	208	CHINA	Jane W. Newburger	770	USA
2	Jane C. Burns	114	USA	Mccrindle, BW	566	CANADA
3	Ying-Hsien Huang	96	CHINA	Ho-Chang Kuo	564	CHINA
4	Adriana H. Tremoulet	89	USA	Jane C. Burns	483	USA
5	Surjit Singh	67	INDIA	Anne H. Rowley	318	USA

### 3.4. Distribution of journals and co-cited journals

**Figure 5:**
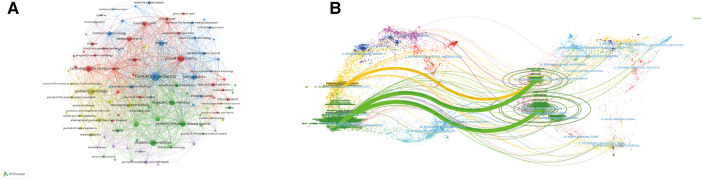
Analysis of journals. (**A**) Visualization of journals. (**B**) Biplot overlay of KD studies in children.

A total of 1,133 journals worldwide published articles in pediatric KD. As shown in Table 4, the most prolific journal in the field of pediatric KD was FRONTIERS IN PEDIATRICS (IF 2.6), with 201 publications. JOURNAL OF PEDIATRICS has an impact factor of 5.1 and is a high-quality journal with many publications. Table 5 shows that the journal with the highest number of co-citations is CIRCULATION (*n* = 2006), which has an impact factor of 37.8. Four of the top 5 co-cited journals have a JCR partition at Q1, among which LANCET (IF 168.9) has the highest impact factor, indicating that many studies on children's KD are based on excellent results, ensuring that the studies are more scientific validity and reliability. Among the journal co-occurrence graphs (Figure 5A), FRONTIERS IN PEDIATRICS is the most significant node, mainly with close cooperation with FRONTIERS IN IMMUNOLOGY, PLOS ONE, and BMC PEDIATRICS. The biplot overlay of journals (Figure 5B) shows the distribution of relationships between journals, citing journals on the left and cited journals on the right. The connecting lines between citing and cited journals indicate the existence of communication and liaison relationships, and the different nodes indicate the disciplinary categories covered by the various journals (27). The yellow path indicates that papers published in molecular/biology/genetics are frequently cited in molecular/biology/immunology journals. The green path indicates that papers published in molecular/biology/genetics/health/nursing/medicine are frequently cited in medical/clinical journals.

**Table 4:** Top 5 journals in terms of number of publications.

Rank	Journal	Publications	IF (2022)	JCR
1	FRONTIERS IN PEDIATRICS	201 (4.12%)	2.6	Q2
2	PEDIATRIC CARDIOLOGY	112 (2.30%)	1.6	Q3
3	JOURNAL OF PEDIATRICS	103 (2.11%)	5.1	Q1
4	CARDIOLOGY IN THE YOUNG	95 (1.95%)	1	Q4
5	PEDIATRICS INTERNATIONAL	93 (1.91%)	1.4	Q4

**Table 5:** Top 5 co-cited journals in terms of total citation frequency.

Rank	Co-cited journal	Citation	IF (2022)	JCR
1	CIRCULATION	2,006	37.8	Q1
2	JOURNAL OF PEDIATRICS	1,629	5.1	Q1
3	PEDIATRICS	1,535	8	Q1
4	PEDIATRIC INFECTIOUS DISEASE JOURNAL	1,297	3.6	Q3
5	LANCET	1,063	168.9	Q1

### 3.5. Analysis of keywords

**Figure 6:**
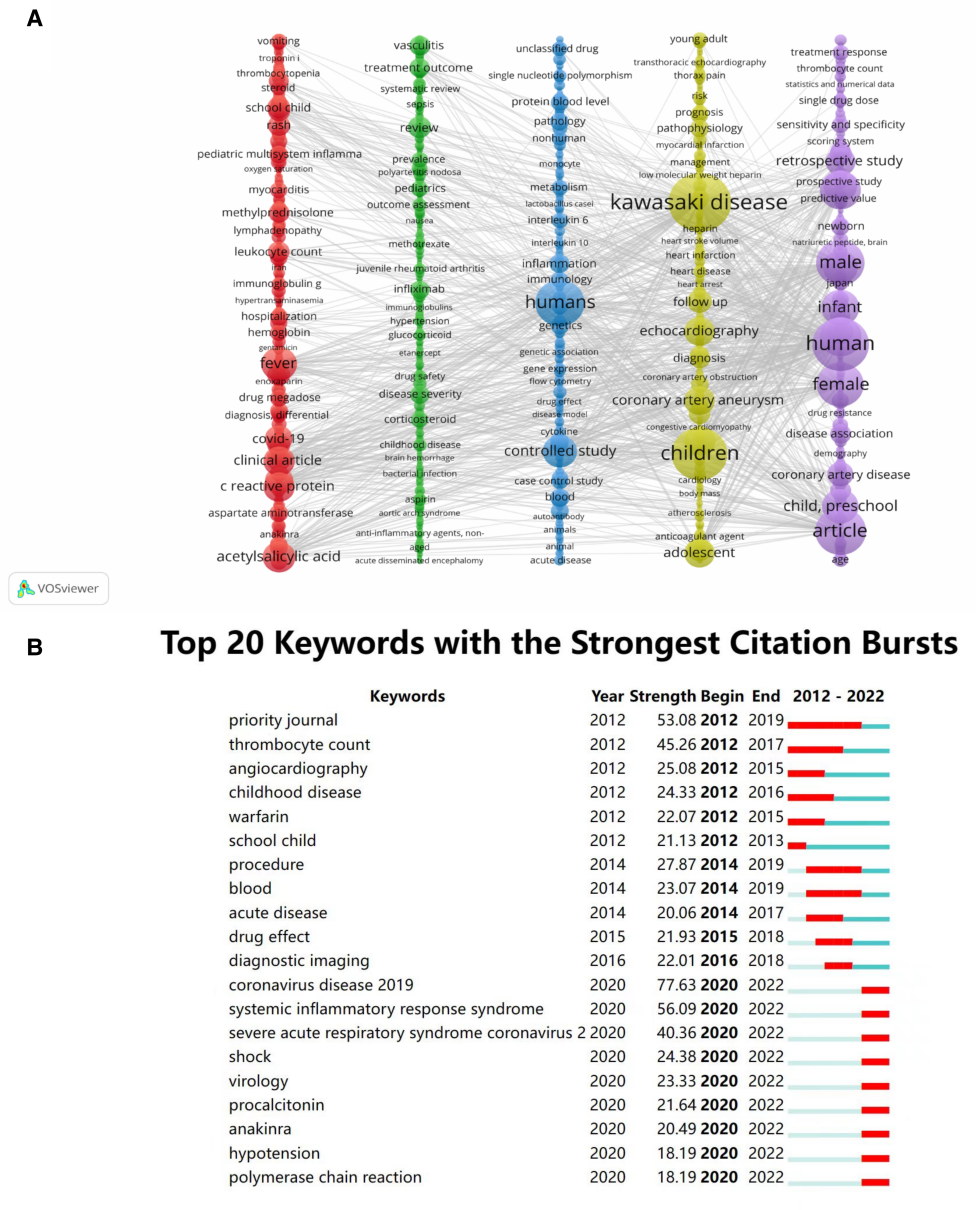
Keyword visualization. (**A**) Keyword VOSviewer clustering diagram for children's KD studies. (**B**) The top 20 keywords with the highest burst values in children's KD studies.

The top 20 keywords with the highest frequency and centrality are presented in Table 6 by analyzing the included literature. Kawasaki disease (*n* = 4,107) and children (*n* = 3,224) had more occurrences associated with including both in the search subject terms, reflecting the immediate relevance of the included literature to the study topic. In contrast, immunoglobulin (*n* = 1,656), fever (*n* = 1,182), major clinical study (*n* = 1,158), priority journal (*n* = 1,023), and acetylsalicylic acid (*n* = 1,016) were the most frequently occurring Keywords. Immunoglobulin (0.94) had the highest centrality, followed by fever (0.73), c reactive protein (0.53), male (0.43), covid-19 (0.37), and female (0.34). Cluster analysis and visualization were performed using VOSviewer, as shown in Figure 6A, and five clusters were finally obtained. Cluster 1 (red) mainly includes the manifestations and treatments of KD in children, such as vomiting, thrombocytopenia, steroid, rash, fever, acetylsalicylic acid, etc. Group 2 (green) mainly deals with drug selection for treating KD in children, such as methotrexate, infliximab, glucocorticoid, etanercept, corticosteroid, etc. Group 3 (blue) is mainly related to the progress of KD in children in pathology, immunology, and genetics, such as single nucleotide polymorphism, pathology, metabolism, interleukin 6, etc. KD-related cardiac diseases, such as transthoracic echocardiography, myocardial infarction, heart arrest, coronary artery aneurysm, etc, dominate group 4 (yellow). Group 5 (purple) mainly included KD-related demographics, such as statistics and numerical data, sensitivity and specificity, human, male, female, etc. A total of 20 co-occurring words were obtained through the burst detection analysis of the keywords. As shown in Figure 6B, the keyword with the highest burst intensity was systemic inflammatory response syndrome, with a score of 56.09. The second was the priority journal (53.08), which lasted the longest time. The third is thrombocyte count (45.26). Angiocardiography, childhood disease, and warfarin were the first outbreaks in 2012. The keywords Coronavirus disease 2019, systemic inflammatory response syndrome, severe acute respiratory response syndrome, severe acute respiratory syndrome coronavirus 2, shock, virology, procalcitonin, anakinra, and polymerase chain reaction appeared first in 2022 among outbreak words.

**Table 6:** The top 20 keywords with the highest frequency and centrality of occurrence.

Rank	Keywords	Occurrence	Rank	Keywords	Centrality
1	Kawasaki disease	4,107	1	Immunoglobulin	0.94
2	Children	3,224	2	Fever	0.73
3	Immunoglobulin	1,656	3	C reactive protein	0.53
4	Fever	1,182	4	Male	0.43
5	Major clinical study	1,158	5	Covid 19	0.37
6	Priority journal	1,023	6	Female	0.34
7	Acetylsalicylic acid	1,016	7	Major clinical study	0.32
8	C reactive protein	925	8	D dimer	0.31
9	Complication	911	9	Abdominal pain	0.26
10	Coronary artery aneurysm	890	10	Diarrhea	0.25
11	Echocardiography	855	11	Thrombocyte count	0.24
12	Adolescent	844	12	Acetylsalicylic acid	0.23
13	Retrospective study	801	13	Vomiting	0.19
14	Coronary artery disease	591	14	Coronary artery aneurysm	0.18
15	Follow up	580	15	Ferritin	0.18
16	Risk factor	548	16	Headache	0.18
17	Covid 19	544	17	Genetic predisposition to disease	0.17
18	Clinical feature	543	18	Coronary aneurysm	0.16
19	Diagnosis	506	19	Coronary angiography	0.16
20	Erythrocyte sedimentation rate	469	20	Myalgia	0.16

### 3.6. Analysis of references

**Figure 7:**
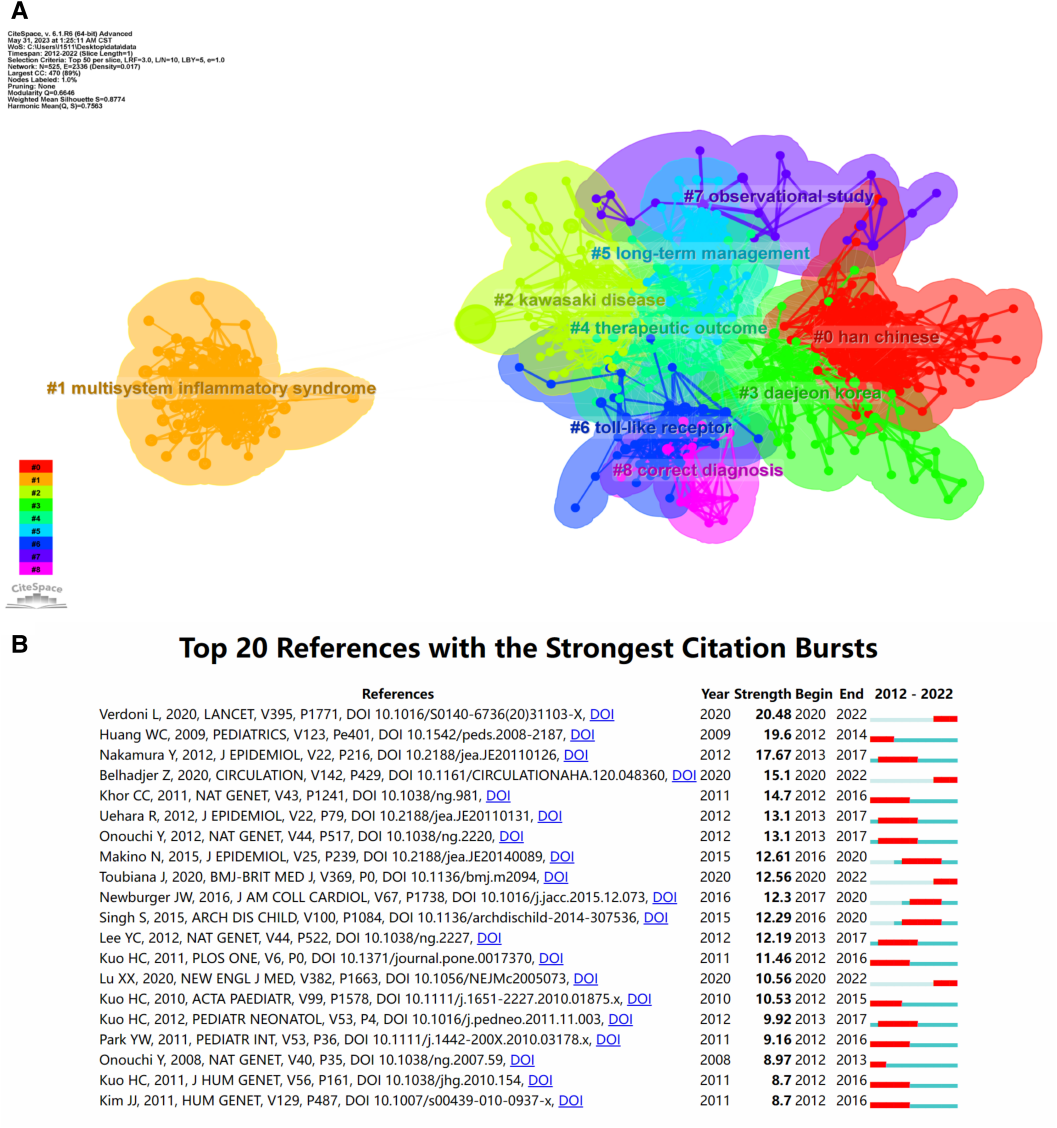
Visualization chart of references in the field of children's KD. (**A**) Reference co-citation network for clustering of headline terms. (**B**) The top 20 references with the highest citation burst values.

Co-cited references are two or more articles that appear in the reference list of other literature simultaneously, reflecting the citation relationship between works in the literature. The ten most cited papers are shown in Supplementary File. Of these, Diagnosis, Treatment, and Long-Term Management of Kawasaki Disease, A Scientific Statement for Health Professionals From the American Heart Association (*n* = 456) was the most frequently cited. CiteSpace with the setting node type = cited reference was used for the co-cited reference clustering analysis, and other parameters were set to default values. We obtained a total of 9 clusters that demonstrate the research themes in KD (Figure 7A), focusing on the etiology of KD, diagnosis and treatment, and long-term management. Figure 7B shows the top 20 references with the strongest citation bursts, with citation bursts reflecting the references of interest to researchers at a given time. Figure 7B shows that the top 20 documents with the strongest citation bursts have burst values that fluctuate between 8.7 and 20.48. “Verdoni L, 2020, LANCET, V395, P1771, DOI 10.1016/S0140-6736(20)31103-X” (28) has the strongest citation burst (20.48). A total of four co-citations have recently burst.

## 4. Discussion

Although research on KD continues to intensify, its rapid global prevalence has continued. In 1970, the prevalence in Japan was only 10.1/100,000 and increased to 359/100,000 in 2018. In Australia, the prevalence in children under five years of age increased from 2.82/100,000 in 1980 to 9.34/100,000 in 2009 (29, 30). In Japan, the United States, and China, KD has replaced rheumatic fever as the leading cause of acquired heart disease in the pediatric population (31). It has been suggested that early treatment with IVIG within four days will reduce the incidence of CAA and IVIG resistance (32). Early diagnosis and treatment of KD are very important for children's health.

This study jointly used Citespace (6.1.R6), VOSviewer (1.6.18), and the online bibliometric website (https://bibliometric.com/) to analyze the WOS, PubMed, and Scopus databases from 2012 to 2022 for 4,867 articles on children with KD literature for visual analysis. The influence and contribution of countries, institutions, authors, journals, and cited literature in pediatric KD were demonstrated. The current research hotspots and future trends are also reflected by analyzing keywords.

Although scholars have been studying KD for more than 55 years, the total number of publications in the pediatric KD literature has continued to grow, as seen in the last ten years of data, especially at a significant rate after 2019. We analyzed this concerning the global epidemic of the novel coronavirus 2019, where groups of children infected with the virus developed symptoms indistinguishable from those of KD, sparking the interest of scientists. 935 publications in 2021, which is 3.55 times more than in 2012, breaking the record for the number of publications. This indicates that research on KD in children is up-and-coming and that the field is extremely attractive to scholars worldwide.

China, the United States, and Japan are fruitful in the field of pediatric KD research, and they are all countries that are highly affected by KD and have a large patient base, thus prompting clinicians to pay more attention to the disease. Among the top 5 countries, the U.S. has the highest centrality, and the centrality of the remaining four countries is less than 0.1, indicating that the U.S. has a significant influence in this field and other countries are less involved in international exchange and cooperation. China occupies three seats each in the ranking of institutions in terms of the volume and centrality but is dominated by research institutions in Taiwan, indicating the unbalanced nature of domestic research. The U.S. institutions need to be sufficiently dominant in terms of the volume of publications, and it is more critical to increase article output while working closely together to provide better references and guidance for global physicians to intervene in children with KD.

Ho-Chang Kuo from Chang Gung Memorial Hospital, the author with the highest number of publications, has studied more on the diagnosis of KD and KD-related coronary artery lesions (CAL) (33–35). Among the cited authors, Jane W. Newburger from Harvard University was cited 770 times, the largest node in Figure 4A. He has conducted several influential systematic reviews of KD in children (36, 37). Jane C. Burns, a top 5 author in both the number of publications and total citations, has proposed that infliximab is a safer and more effective treatment than a second IVIG infusion for patients with KD resistant to IVIG in a randomized, multicenter randomized trial (38).

The top-ranked journal, FRONTIERS IN PEDIATRICS (IF 2.6 Q2), published 201 articles on children with KD. Four of the top 5 journals have an IF less than 3, and only one journal has a JCR division in Q1. Researchers should aim to improve the quality of articles to meet the requirements of high-quality journals and improve the overall academic standard in pediatric KD. The JCR partition of the top 5 cited journals is located in the Q1 region, and CIRCULATION (IF 37.8 Q1) is the most cited journal with the strongest influence and recognition in the field. The articles in LANCET (IF 168.9 Q1) are heavily cited, reflecting the reliability and credibility of the research results.

The most cited article was published by Brian W. McCrindle in 2017 in the journal CIRCULATION. The article (39) updated the diagnosis, treatment, and long-term management of KD for the revision of the 2004 American Heart Association guidelines on KD, and it became an essential reference for clinicians in this area of practice. The article (28) with the highest outbreak intensity was published in 2020, in which Verdoni, L et al. concluded that the SARS-CoV-2 outbreak was associated with a high incidence of severe KD through a cohort study of children with severe Kawasaki-like disease in the Italian SARS-CoV-2 outbreak, noting that similar outbreaks of Kawasaki-like disease occur in countries where outbreaks occur. The article (40) with the second highest outbreak intensity came from Yosikazu Nakamura, which explored the epidemiological characteristics of KD in Japan from 2009 to 2010 and found that the incidence of cardiac lesions and cardiac sequelae during acute KD was higher in the infant and elderly groups. The proportion of patients with giant coronary aneurysms did not decrease significantly with lower KD incidence.

From the analysis of the results, we believe that the “national-institution-journal” model can strengthen international cooperation in pediatric KD and promote more fruitful results in this field to benefit children worldwide. (i) East Asian countries (China, Japan, South Korea) have more clinical cases and published literature, and North American and European countries (USA, Canada, UK) have rich experience in cooperation and exchange. The academic barriers between countries should be broken down, and each country should learn from the other's strengths and strengthen the dialogue and communication between scientists from both sides through frequent study visits and training to form an authoritative international consensus on diagnosis and treatment. We should also build a global platform for sharing pediatric KD cases and research results so that pediatricians can have a more intuitive and comprehensive understanding of KD. (ii) Influential pediatric KD research institutions are unevenly distributed internationally and domestically; they are mainly from China and the United States, while Chang Gung University and its related institutions occupy a significant position in China. These institutions can take advantage of their disciplinary strengths and establish their research clusters from within China, gradually forming a regional scale to coordinate the characteristics and diagnostic and treatment difficulties of pediatric KD in the region, which is more conducive to inter-country exchanges. (iii) Journals that publish many pediatric KD research results are pediatric-related journals with solid specialization. They can regularly invite influential researchers in the field of children's KD to present their latest research or add a KD column to bring together the research results of scholars worldwide. They should also keep looking for new faces in the manuscripts which have made outstanding contributions to the field and build contact databases to help authors communicate with each other more deeply and conveniently.

## 5. Hotspots and frontiers

The frequency and centrality of keywords suggest that “immunoglobulin” is the focus of research on KD in children, but what makes this primary treatment a hot topic? Our review of the literature revealed that researchers have focused on the area of “immunoglobulin resistance and complementary therapy”. The clustering of keywords shows that “KD pathogenesis” and “cardiovascular complications” are also the focus of research in this area. We believe that “KD pathogenesis”, “immunoglobulin resistance and complementary therapy”, and “cardiovascular complications” are the hot spots for research on KD in children.

(1) Pathogenesis of KD in children. There are three main hypotheses about the outbreak of KD: infectious, genetic, and immune. KD has a higher incidence in winter and spring, with more aggregated cases, and has the same characteristics as viral infections (5). The age of onset is concentrated in children between six months and five weeks of age. Before six months, the passive transmission of maternal immunoglobulin protects in early childhood, while after five years of age, the immune function becomes more refined, suggesting that KD is influenced by immune response characteristics (41). Siblings of KD patients have ten times the risk of disease compared to the average population, and children of KD patients have twice the risk of disease compared to the average population, suggesting that genetic factors are involved in the pathogenesis of KD (39). Researchers generally accept that KD occurs mainly due to the invasion of pathogens into genetically susceptible individuals, activating multiple immune cells, which leads to abnormal activation of the immune system and the waterfall release of inflammatory factors in the body. In the acute phase, KD is mainly a disorder of the immune system, and inositol 1,4,5-trisphosphate 3-kinase C, a polymorphism, is the primary initiating factor. Suppose its negative regulation of T cells is lost under the stimulation of bacterial superantigens (staphylococcal toxic shock syndrome toxin and streptococcal septic toxin). In that case, the abnormal activation of T cells increases, activating B cells and producing a large number of inflammatory factors such as IL-6, TNF-α, TGF-β and other immune mediators, which leads to vascular inflammatory injury and thus increases the severity of KD and the risk of CAL (42). As the understanding of KD continues to advance, scientists have found no single indicator for diagnosing KD, and physicians rely primarily on the clinical features that constitute the epidemiological case definition to confirm the diagnosis. However, conditions such as measles, staphylococcal and streptococcal toxin-mediated diseases, systemic onset juvenile idiopathic arthritis, drug allergic reactions, and especially multisystemic inflammatory syndrome overlap with symptoms of complete or incomplete KD (43, 44). The continuous proposal and updating of pathogenic mechanisms may provide an essential reference for identifying and clarifying KD.
(2) Immunoglobulin resistance and supplemental therapy. Since the addition of IVIG to the KD treatment regimen, the incidence of CAA has decreased from 25% to 3–4% and mortality from 1 to 2% to 0.1% (45). However, 10%–20% of KD patients do not respond or develop persistent fever within 36 h to 7 days after the first IVIG injection, called IVIG resistance (46). It has been shown that children with IVIG-resistant KD have a higher risk of CAL (47). Liu, GY et al. (48) concluded by a meta-analysis that severe anemia, hypoalbuminemia, decreased baseline platelet count, and elevated ESR, total bilirubin, ALT, and neutrophil percentage predispose KD patients to IVIG resistance. In contrast, males, hyponatremia, and elevated AST and CRP levels are risk factors for IVIG resistance in Asian Mongolian populations. 65% of infants under six months of age who received timely IVIG therapy still developed CAA, presumably associated with auto-resistance. Corticosteroids are currently recognized as the primary treatment for IVIG-resistant patients due to their anti-inflammatory and immunosuppressive effects (49). Methylprednisolone is the most commonly used corticosteroid, but prednisolone is recommended by the Japanese medical establishment. The following are also currently reported alternative therapies for IVIG resistance (50–52). The biological agents' infliximab and anakinra reduce the systemic inflammatory response and shorten the duration of fever by inhibiting TNF-α or IL-1 expression. Plasmapheresis can directly reduce inflammatory cytokines in the blood to alleviate the inflammatory response. Cyclosporine interferes with the 1,4,5-trisphosphate 3-kinase C/calcium-regulated phosphatase pathway to inhibit T-cell differentiation and treat refractory KD, but efficacy remains controversial. Methotrexate reduces IL-1 and IL-6 levels in KD patients, and some studies have shown that oral methotrexate significantly reduces the characteristic clinical symptoms in KD patients.
(3) Cardiovascular complications of KD in children. Cardiovascular complications of KD mainly include CAD, valvular lesions, coronary aneurysms, giant coronary aneurysms, coronary stenosis, and acute myocardial infarction (53). In the 25th national survey in Japan, CAD accounts for 6.52% of all types of complications, and 2.76% of patients are left with cardiovascular sequelae (54). The following pathological changes are seen in the arterial wall of the heart with a history of KD: inflammatory cell infiltration, destruction of the intima and media, the proliferation of intimal myofibroblasts, and replacement of myocytes by fibroblasts and connective tissue (55). Patients who develop CAA are at high risk for later coronary thrombotic or stenotic lesions, which may eventually progress to myocardial ischemia, infarction, or even death. A consensus (56) of UK and US experts on patients with coronary complications of KD states that the basis of long-term management of CAA is prevention, including regular checkups used to detect thrombosis or stenosis promptly and the adoption of healthy daily habits and lifestyles (e.g., alcohol cessation, smoking cessation, low-fat diet, exercise). Guideline (56) also recommends that all children with a history of KD and a risk class greater than or equal to 3 [small aneurysm (2.5 ≤ Z score < 5)] be included in adult cardiac follow-up management without interruption at age 16 to 18 years.

The combination of the keyword burst value analysis and the cluster analysis of the reference title terms concluded that the frontiers of KD research in children include three areas: disease-related (“multisystem inflammatory syndrome”, “coronavirus disease 2019”, “hypotension”), treatment-related (“procalcitonin”, “anakinra”) and pathogenesis (“polymerase chain reaction”). The related literature aims to gain a deeper understanding of the impact of KD in the pediatric field, to avoid clinical misdiagnosis, and to achieve proper and comprehensive treatment.

## 6. Advantages and limitations

No researcher has published a bibliometric analysis of KD studies in pediatric populations. This study uses Citespace (6.1.R6), VOSviewer (1.6.18), and an online bibliometric website (https://bibliometric.com/) to summarize comprehensive information and hot frontiers in KD from a bibliometric analysis perspective, providing a meaningful reference for research in this area. This study is not perfect and has limitations. First, there may be subjectivity when manually screening the included literature. Secondly, we only retained literature whose language was English. Finally, VOSviewer and CiteSpace do not allow for advanced statistical analysis, which may introduce statistical bias. However, these limitations do not affect the extraction and analysis of comprehensive information on the field of children's KD.

## 7. Conclusion

The bibliometric analysis of this study presents comprehensive information on articles in the field of pediatric KD in the last decade. We find that research on pediatric KD is in a phase of rapid development, with an increasing number of countries, institutions, and researchers taking a great interest in pediatric KD. Although results are emerging, the pathogenesis of pediatric KD, cardiovascular complications, and alternative therapies for IVIG-resistant individuals are still pressing issues that will be at the forefront of future research. We summarize and propose innovative measures for global collaboration in pediatric KD. We hope that the “country-institution-journal” collaboration model will help researchers and clinicians achieve more extraordinary breakthroughs in the field.

## Data Availability

The original contributions presented in the study are included in the article/**Supplementary Material**, further inquiries can be directed to the corresponding author.
